# Supplementary material for: Preparation of Fe3O4@polyoxometalates Nanocomposites and Their Efficient Adsorption of Cationic Dyes from Aqueous Solution
Source: Nanomaterials (Basel). 2019 Apr 23;9(4):649. doi: 10.3390/nano9040649 (PMC6523850; doi:10.3390/nano9040649)
Supplement: Supplementary file 1 [file nanomaterials-09-00649-s001.pdf]

Supporting Information

Preparation of Fe_3O_4@polyoxometalates Nanocomposites and Their Efficient Adsorption of Cationic Dyes from Aqueous Solution

Jie Li, Haiyan Zhao, Chenguang Ma, Qiuxia Han, Mingxue Li * and Hongling Liu *

Henan Key Laboratory of Polyoxometalates, Institute of Molecular and Crystal Engineering, College of Chemistry and Chemical Engineering, Henan University, Kaifeng 475004, China; hedalj@163.com (J.L.); 15890943739@163.com (H.Z.); 104753160773@vip.henu.edu.cn (C.M.); qiuxia_han@163.com (Q.H.)

* Correspondence: limingxue@henu.edu.cn (M.L.), hlliu@henu.edu.cn (H.L.)

Thermogravimetric analyses of Fe_3O_4, **1**, **2**, Fe_3O_4@**1** and Fe_3O_4@**2** were performed under a nitrogen flow (Figure. S1a, b). It turns out that the weight ratio of 1 and 2 in the synthesized nanocomposite is 96.68% and 96.50%, respectively. It should be noted that the loss of Fe_3O_4 might be attributed to the loss of its surfactant attached during the synthesis process.

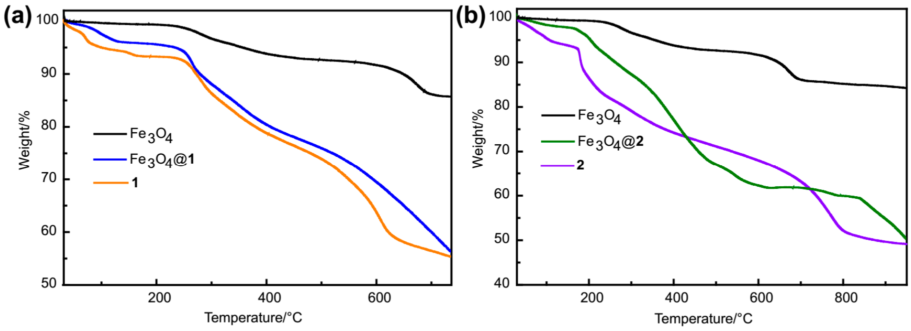

Figure S1. Thermogravimetric analyses of (**a**) Fe_3O_4@**1** and (**b**) Fe_3O_4@**2**.
